# Supplementary material for: Transcriptome Profile Analysis Reveals that CsTCP14 Induces Susceptibility to Foliage Diseases in Cucumber
Source: Int J Mol Sci. 2019 May 26;20(10):2582. doi: 10.3390/ijms20102582 (PMC6567058; doi:10.3390/ijms20102582)
Supplement: Supplementary file 1 [file ijms-20-02582-s001.zip › ijms-505688/Supplementary Files/Supplementary File 1.docx]

**TCP binding site GGNCCC analysis of CsNBS-LRR genes**

**Note:** The No.8 was the candidate target gene of CsTCP14, which harbored three binding cite GGNCCC in its promoter.

**1. Csa2M020860.1:**

**Promoter：**

AGCTTTTTTGTTGGCATGCCTTCAAAAAAAGTCATCCATCTAGTAATTATTTAGACCTTTCAGAACGTGCTACAAGGCATTGTAAAGGTCATCCTTTGGCTCTTGTTGTTTTGGCTTCTTTCCTTTGTGGCAGAGATCAAGCAGAATGGAGTGGTCTATTAGATGGATTTGAAAACTCTTTGAGAAAAGGTATTAAAGATGTTCTTCAATTAAGTTTTGATGGGCTGGAAGACGAAGTAAAGAAATTTTTTCTTGATATTTCTTGTTTACTCGTGGGTGAGACAGTTACCTATGTTAAGAAAATGTTGAGTGAATTCCATTCGATTCTGGATTTCAAAATTAGCAATCTGAGGCATCTTTCACTTATTAGGATGGAAGAATATGATGATGATAGGGTGCAAATGCATGATTTAATAAAACAAATGGGTCATAAAATAGTTTATGATGAATGTGGTGATGAGCCTGGAAAAAGGAGTAGATCAGGTTGGAGGAGGACATTCTGGAGGTGTTTAGTAACAATTCAGTAAGTTGGTGTTACCTGAATTAATTGTCTTTAATTTAAGTAATTAATTTTCCAAGACTTTCAGATGATGAAAAATGTTTGTGAAAGTAATGTTAAATTATACAAAATGTATGACTTCAATTTGTAGGGAAGCGATGCAGTGAAAGGCATAAAGTTGGTGTTTATCTGATCCCACAAGGGTCATAAACGTGAATCCAGAGGCATTTAGAAGCATGAAGAATTTGAGAATACTGATTGTTGATGGAAATGTGAGGTTTTGCAAAAAAAAAAAGTATGTACCGAATGGGTTAAAGTGGATAAAATGGCATAGATTTCCTCATCAAACTTTACCCTCATGCTTTATTACAAAAGATCTGGTTGGACTACATTTGCAACATAGCCTCATCACTAATTTCGGAAAAGAACTGTATCTTAACAACTGCCCAAATTTAAAAACCATTCCTAAGTCATTTCTTTCTCTTCCTAACCTTCTTACCTTCAACCTCCACCATTGTTTAAACCTTAAAAAGATTCCAAGTAGCTACATTTCATGGGAGCTAGTGTCTTGAAAATTTTGATCTTTCTCACTGCCAAAAGCTTGAAAAAATTCCTGACTTCTCTTCTGCATTAAACCTTAAAAGCTTGTCTCTAATACAATGCACAACCTTGAAAAGCTTCCAAGCTACCTCAAGTTAAAGTCTCTTACAGATTTAGATCTCTCTGGTTGTCGTAAGCTCGAAACGTTTCCAGAAATTGATGAAAACATGAAATCCTTAGAAAGGTTGAGGTTGTCTTATACGGCCATAAGGAAGCTACCTTCGTCAATTGAACACCTTACTAATCTTCAATATATTAAATCTTAAACGTTGCACAAACCTCATCTTCCTTCCTTGTACAGCTTATAATTTGTTTAAGAGTCTTTGCGGGCTTAGTCTTTCTGAGTGTTCTATTGAAATGCGTTCCAAAAGTTATAAAAACTATGAAAATTATGAAAACTTCTTCGACTTGGGAGTTTTTCCATTCACGAGTTCCAAAAGAAATCTTATTTTTCGAACATTTGGAGTTGCTGGATCTTAAAGGTTGCAATATATCAAATGTTGATTTTCTGGAAAATTTATGTAATGTAGCTCTATCCTTAACTAGTATAGTCTTGTCAGAAAACGAATTCTGTAGTCTACCTTCATGTCTCCATAAGTTTATGTCCTTGCGGAATCTCCAATTAAGGAATTGCATGTTTCTTCAAGAAATTCCAAACCTCCCTCAGAGTATACAAATAGTAGATGCCACTGGTTGCATATCGTTGAGAAGAAGTCCAAACATTATGTGGACATAATATCAGGCGGGCAGGTTCATCTCTTACCAATTCATTTTCTTTGTTCATATCAAGTTCTTGTCTTTTATACTAACATTCCATGCATAATATATATATATAATTCTTATTCCATAGGTCAATGTTAAATGGAGAAGGCGTTGTTATATCTTCAGGGAATTCGTTCTA

**2. Csa2M021510.1:**

**Promoter：**

AGAAGATGATCCACTTGCTCTATCCATCTGATTACATGACTTGTTATATTTTGCAAATAGTTTGTAATATTCCTAATTTGATCATGCACATGTTAATGCAACTCTAATAAAAAAATTGTTGTTATTGAATGTTAAACTTTATTGTTTTATAGGTGCAATGAATATTTTTAAAAAAATAAATTGAATTATAGTTGCAATAAATATTTTTAAAAAATAAATTAATAATTGGGGTATTTTTAAAACTATGAGATTTTACAAAATATTTACAACATATAGCAAATTTCATCACAGATAATCATACTACTATAGTGATAGAAGACTATAATTGATATAATCCAAATTTTGCTATAATTCGTAAATATTTTAATTTATTTTGTTATTTTTTACAATGCTCCTTAAAAATTTTATACATGATAAATTAATTTATACTTTTTTGCTATAATTCGTAAATATTTTAATTTATTTTGTTATTTTTTACAATGCTCCTTAAAAATTTTATACATGATAAATTTAGGATGTACAATTAACAAAAAAAAAAAAAAAAAAACAAACAAACAGATTAGCTAAGCGGAATTCAAAACCAACACCCTAAACACAAGCATTAGAAATCCAGATTACCAATGTCTACACTCAATTCTTGCACTCCACATCTGGAAATTTTCAAGCATAAAAACTTCATCAAATAAGCCCACAGTCATTTAATTACCATACATTTGATTACAAACTTGCACGCTAAATACCCCTTATATGTTATCTAAAGAAATCACGTTAACGGGGAGGTAAGAGAAAAGAAGATTTCATATTTTAGTCCGTCATTTCGTGCTTTTTTAAAATTTATATTTGTTTTCTCCTTTTCTTTTTCAATAATGATTTTCAAATTAGTTGTAAAAACATTTGAACTCATCGTAAAATGTCAAAATATTTATAGAAAAATTTCTAAATGTCATCGATGATAGTCAATGATATACTACAATGATGGTAATTTATCGGTAACTATCATTGATAGAAATATAAAATACAAAATTTTGTTATAGCCGATAAATATTCTAATTTATTTTGATATTTTAAAAATGTCCCTTTACAAAACTAATTATTTTCTAAGTTTAATATTCAAACCATCACTTAATTAATTTTTTAAAATAATTAAAACAAAGTAGATAACAATAATAGAAATTTATGGGCGAAACCGTGTGTTCATATGCTTCATTTTCAAAAACTAATAAACAAATACCAAAGGTTAAACGAGCCTTAGAAACAATAAACGATTGGTTCGATATGGAATTATTGCACTTTTCGTTTTCGTCATGTTCTGTCTCCACTCCCTTGGGCTGGATATAATAAAATGCTAAGGAAAAAAGGGTTTTGAAAATTAATTATTTTGCAAGTTTAATTTTCAAACAAACACTTAATTTTTTAAACTAACTGAAACAAAGTAGATAACAAATCATAGAAATTTATGGGTGGAGGGTGTGTTCATATGCTTAATTTTCAAAAGAAAAAAAAGAAGAAACAAACAAACACCAAAGGTTAAAGGAGCCTTAGATTTTTTGGTTCAATATGGAATTATTCCACTATTCGTTATATGCTGTCTCCACTCCCTTGTGATGGATAATTGCTTGGTTGGATGTAGTTCGAGTTTATTTTCTTGCTTTGATTCTCTAGCTCGTAATTTTAGGAATTATTCTGAGATTAAAAAAGAAAGTGTTTCATGAGACTTCGTCAGATTCTGTCTGTGTTTATTAATAGAGAACTATTTAATTTATACAGTGGTGAAAAATATATTTAAATATCATCGTTTCCAAGTGAAAATTTAATAAGAAGTGAATAAAATAGTATTTTAACTAAAAATTAATTTTTTTAAAAAGGAAGAAGACTTGGGGAAAACGTTTGTTTCAAAGTCAATGAGAAAAAGACAGAGAGGAAGGTTGTTGGAATCAAGATGCCACCAAAAATATGACAGACAAAATGGAAGAAAATGAGAAACAGAGAGAGGTCTCCAAGGTATTGATAAGGTATTCACTTATATATCTTCTACTTTTCCATTCCTCAAT

**3. Csa2M021540.1:**

**Promoter：**

CCACCTGCTTGATTCATTTATCTTTTTTTGTCCTAGTGATTTAATTAAGAGGTGGGCTTGATGGATCCATCATAATGGGCTATTGTTTCTTCATATGATGGCTTTATATAACATAATATAATGGTCACTTGATGATTTGTGTTGAAAAAGACAGTGATGTTAACAATGTGAACTATATCAATAACAATTATTATTTGTTAATTTAACTTATTAAAAGTAAAGTTCAATCAATTTGATTATCAACATATTTCAGGTTCGTAATATGCGCATTTGTTGATTTATCAAGAGATTCTAAATTCATATTTGTTGTACGTACTACTTGTAAACTTTCTCTGATCTCTGTTTGTGTTAATTGTTTCAAAAAACAACACATGGGAGATTGAAACACGATCTTTTGAAATTAGTGACAGAGTAAAAAAATTCATATAAGGAATACTATTTTTTATACATATGAACTAAGATTGACAACCGGGTCATGATCTATTAACCTCGTTCCCATCCCCTTGAGGGTATTTAACCTCATATCCCTGCCTCGGCCCGTGGGGATGGAGGCATGGATTCCCTATTGAGGGGAACAAATCCGCGTGGTGATCCATTCCTAGATTCTTAATTCTTAAATTAATTAATGCAATATCTATTTACTTACTAATTTTAAAATTTAAGGTTAAGTTAGAATTAAAAGTTTGTAGACAAATTAGAAATTTGACTAAAACTTTGGACATTTTTTAGACACAAATTTAAAAGTTCAAACAACTTGTAACGTAAAACCATATAAATATGAAAACTATTGTAATATCTAAATTAAGTAATATTAATTACATAATTATAAAAAATTCTAAATATATAAGCAAGTTTTAGATATTTAAAACCAATATTTACGGCATATTTAACAAAACAACTCATCTCCTTCCAAAGAAAATAATTAAAACCCATATTTAAGTAAAACTATTAAATAAAATTTAAGGTTAATTTTCATAAATATAATAAAACATTAAAATATTTACGGCATATTTAAAAAAATCAAAATGTCTATGAAGCCAATTTTTTTAAAAAATATTTCACGTTTATTCTTCTTTTTCATATTTTTTTTCTATATTCTTTCTTCTGTAATTTTTCTTTTACATATATTTTTTGAAATCATGATGTTTGTTTTCGATCAATATTCATAAATATCGTATCATTTTTAGAGTGTTATTTGGTTCAAGATCCTATACCAAATATAAAAGATCTTGAAAAAAAATCGTTGGAATATAGGATTTAGATGTGGGTATAGCCAAATCTAAATGATTTAAAAAAAAAAAACAAAACAATCTTGAAAAAAAATCGTTTAAATTTGAATAGCCAAATCTAAACCATGGTTGAAAAAAATTGTGTAACTGTGTAGCCAAATCTAATGGCATGTTGACTGGAGCACTTTTGGTATTTTCTATTGTAGGTCTGTGTAAATATTTAGTCGATTTGTTATATATTTTTTTTGAAAATTCTCTAAAATTTAAATCATGTAAATTAACCTAAATGACATAAAGATTCTAAAAATATCTAAAAAAAATAGTAAAATTTTTAGATTCTATCAAGAAATAAACCCGTATAGGATTTAAAATTTTATTATATTTTATGAATATTTGAGTTTATTATACAATAGTTGAAGAAGAAGAAATTTGGGATCATGTAGGATACACTTAACTCATGTGCTTTCCATCACCCCCACCGTGTAAAATTTATTTTTGTACAGTGGAGAGATAGAAGTACTTAATCACTGCCTCATTTGAAATGACCCCTAAAATGTTATTAGATATTAAAACACTTCAAATTTAAAACTAAGTTAGCTAGAGAAAAGAAAGACCAAGAGACTTTGGAGGGAAACGTTGTTCAAAGTCAATAAGAAAAAGACAGATCAGGAGGGTTGTTAGAGAGAAGAAGTCACCAAAAAATATGAGAGAAGAACAGAGAGAGAGAGAGGCACCAACTATATTAATAAGGTATTGACTTCTCTAATTCTACTTTTCCATTCCTCAAT

**4. Csa2M022270.1:**

**Promoter：**

GGAAACATTTTTCATTGAATCTCTACAATCCCATCCTCTTCCTGCTACAATCTTTTTACTTTCTGGATTTAGTCTAAGAATTGAAGCTTTACAATATAATATTTCATACAACTCCAGCTATTGAAGCACTAAACTAACCATGAAATTCAAGAATATTTTTAATATGTAGGGTGGGGGATTAGTACGATAAAATATTTTAAAGAGTGTCTTTCTATCTATGGGTAACTTGAAATTATTTGATTTAAAGTTTTAGTATATTGATATGTAAACTATACAACTTAAATTCATATTATTGTATTATGTAATTATTTAATTTTATGCTTTTGATTTTTTTTTTTTTATAAATTGGACATGTAAAATATATTGAATTCATCACTTTTATGTGGTTGAACTTTCATTTTTCATGTTATTATTGCAATACATCATGTTATAAATGAATTACTTTTTGACAATAGTTTGCACAATGGAACCTGGTTCGACTGATCCTATCCAACTATACCTATAGACGTTTCATCGCTCACCGTCTATGGGATACCTCCACAGTTATTTTGAGTTGTCGGAGGACAGAGGCAACATTATGGACAACCCCTTTCAAATTAGCGGATTGTGCCATACTCAGAGGCGGTTGGATTTCTTGGGATTTTTCAGATAAGATTCATGCAGTTGGATTGACACTTGATCACTATTTTGGTTGAGCGTTGGAGATTAGATACCCACATCTTTCATATGTCTTTTGGGAAATGCACAATCATATTATAGGACGTTGCAATCCAATTGGGGTTACCGATGGATGGTGAGTCTGTTGTAGGCTTGTTAACTGGAAGCAAGTCTGTGAAGATTTTTTGGGAGTTCTACTGACATGAAAGGTCAACGATTAAGTCTTCAATGGTTGGCAGAACAATTCAGAGAATTGTCACCAGATGCCGACGTTGTGAGTGTTTAGAGATATGCTCGTGCATATATATTATGCAACTCATTGGAGGTTTTCTGTTTGTTGACAAATTAAATACTTGGTCCACTGTATGTTTCTTCGTGATTGCGAACATACTTGTACATATTCTTGGGGCGCTACATGTCTTGCATGGCTGTATAGAAAATTGTGTCAAGCCAGTAATGTTCGATCTTTAGAAATAGTAGGCCCATTAATGTTACTTTGCAAGTATGAGCATACGCCAAATTCCCTGGTACCACAAGTAGCACTATACGCTCCAATTGATCCACCTTTGAGTTTCGTGTATTATTTTATTTATATATTATTTTTTAATAAAATTTACACTATTATAACTTATAATTAATTTTTTTTACACATTAGATGAAGTGGTGTTCTACGTATACCTACATCAAAATAGTCAGCAAATATGTTGTTGACTTACCGAATGATATTTGATCGACTGATACACCCTTCGGTAGTAAAATATTTGAATGGAAACAACTCTGAAAAACTAATATCAACATCTAAATTGCACAAATCTTTAAGGTCCTTGGATTTGGATAACTCAAACTTTGAAAACTAATATTAGTATTTGAAAACTTGTTTTTACTTTTACAATTTAGCTAAAAATCCAATTTTTTACTCAAGAACAATGAAAGCTACCGTAAGAATTAGAGGGAATAAATTTGTTTTAGAAAACCTTACACTAACAAGCAAACGGTTATGAAAGGAAGTGTTGGTTTTTAAATTTCAGTTTTCTAAAGAAATAAAAACCATACACGTGAACCAAATATGAACTCTACAAACAGACTTCCCACCTTCTTGACTTTTATGCTCCCTTCAATTTAGTTACAATGTGGGAAAATGGGAGTGGTAGAGAAAAGTCTTTGGATGAAATTGGATAGGTTGTGTAGTACTGTATCAATCAAATTCAAAATACGATGCCCCACTTCTTCAATGCGTTTGATTTTCTCTCTTTATCTAATACATTGTTCTTCAATTTCTGCTCTGCATTATTCTTCATAAGTATTGCTATTGGTGTTTTCGATTTTTCTTTTCCTTATTCTGCGCGTTTCTTTCTG

**5. Csa2M022780.1:**

**Promoter：**

AATATTATTGGATTGTTTTGTGATGTTTTAATTCTCCTATTATAAATCATTATTAGGGATATATATATATATATATATTCTAATGTGATAATGGTCCAAACTCTCAACTACTCAAAGGTACTCTCAAATAACCATTACCTCACTCTTACTATCTATTCAATTTTTCATTATCATAATGGTCCCTTACAACTTATAAATACTATACATTTTTTATGCAATAAATGAATCAATATTAGCATATATAATACTCCTTAATTTCTAAGTTTATTTTTGGAGAAAATAAGTTATAAAGATTGAGTCATCTAACCTGTATTTTTAAAATTGTAAAAATAACAAATTTAAAAATAGATTATCATCAGATTATTACCGTCGTATTTGTCAAATTTGCAATATAAAAAAAAAGTGTTATGAGCTGTTTTTTTTCTAAATTTTTTATCATCTAATGCAATTTTTCTTTATTTTTCATCTACCTTTTAAATTATTCAAAATACTTTTAGTTCTTAAATTTCAAAATTGCCAACATTATTTGAGTTTGTTTCAATTTAGTCACCATATTTCAAGATTTCACTTTTATCTTTATTTTTAGTCTTTAGTATAGATGCCGATTAATTAATCAAAATTATGATTGATTAATTAATTTTATTAATTTTTACTATATTTCATCGTTATTAAAATTAAATTTAAAATTTCGTTTCATAATTATTTTAAATGAAGGAACATATACAAATACCAAATAGTGAAGTATGAGTATATAGTAAAAAATTAATAAATTGAGATAAAAACTAAACATTTGTAATCACTAAACTAAACTATATCCATACCAAATTGAAATCAAAACTCAAAGTTTAAAAGGTAATTTAAAACCTAAAGACAAAACAAAATTGGACTGAAACATAGAAAGATAAATAAATAAACCAATGAAGTATAATGTTTAGGATGAGAACAAGGACTTGATTAAATTCAAATTGAGATAAAGATTACCTTTTTAATTATTATTATTTTGGTTCTTTATAAAAACAATAAATTAGTGTGATAATTTTATTCATTCATATTTCTTGTCCTATTCCAAATATTATTTTAGATAGAATCAAAAGAAAAGTTAGAATGTTCCCTTTAACTACTTATATTAAAAGCCCTAATCCCTTCCTTCCCTTCATGGTTTTATATTGTAAAATTGATTGAACCCTTATTTTCTAAAAATAATAAATGAAAGAAAATGATTGAAGTTAGTGGGAAAAATATGTATATATGTATATAGGAAACAACTTAAGTACTTTCATTTTTAAAGTGTAAGAATATAATTCATTATTATTTAGTTTTGAATTGAATGAAATTTTATCAATTACTTACATTGCAAATAACATTTAATTATCATTATATATATAATAGACACACTTACATAGATCATTAATATTTATCTTTAACAGTAGTTTAGTTTAGGCTGAAAAAGTACACAATAAGTTAGATTACTTATAGTAAATTGTTTGTCATGGACTCAACTTTCTTTTCATCTTAGTTATATTTTAAAATGCATTGTGATAAAAAATGATAATGACGACTCTTAAGTTAAATTATAAAAACTAGTAAAGTTGAATTTAAAAAATAGTTCTATATTTTGAAAAGTGGAATGTTACGTAAGAATTGAATTTTATTGACCTAATTTTTACTATTGAAAATTTGAGGTATAATTGTAATTAACAATAATTTTTTATATAAATAATCGTAATTCCATCGATTATAAAGTAAAAGAGAAAAAAAAAAGTAAACATTTTTTAAAACTTTCAATTATAAATTTGTAATCAACTAAACTTTAGCGTCAAGATTTATACAAGAGGAAGAGGCCGAATCATGACCATTTTCCTATCAAGTTCTCCTTTTTCAATGAAGATTCTTCATAAGTTGGAATCTTTCCAAAAAAGAAGCTTAATCAATGGATTTGTATATGAACTGTGAACAATTGAACATCTGTTCCTTGTGGGAAATTCTTGTAAATTGCAACAATAATAATA

**6. Csa3M044470.1:**

**Promoter：**

ATTAAATCGTAAGTTAATTTCATAAATTTTGAGGTTCGTTGTTCCTATTACCTTTTAAAAGTATTTGATATAAGTCATAAAAGAAAAAAAAAAAGTTAAAATGTCAATCTGACCAACGTAAGTAAAAGTTATTCAATTTTAGATTTTGTACTTTTAAGTATCTAACTTTACTTTATATATTTTATCTTAATTTTAGTTCATATATTCTGAATTTATTTATATGTTTTTATACAGATTTTTTTTTTTTATATATAATAACTAGTTTGGATTACATATAATTATTTAACATAAATGACAGCCATAATATGGAATTGTTGTACCTCACAAGAATACGAACTTAAAAGGTAGGTAGTATAATTAATATTTGAAAGCTAAAAGTATTTGAAATTGATTTACAAATTATATATGTATAAACTAAAATTAAATAATTGCTTAAATTAGATGTAAGAGAAATGTAGTCATAAATTATACTTTAATTTGTTGTTACGTTGATTATTAATTATTCAATTACTAAAAATAAACAAGTATTTTAAATAGTAGTTATTAATTATTAAAAATAATTTTTATATTAAATGACTAAAAAATTATAAAAATAATAATTGTACTTACCATGGAAGCTCAAAGTTGATGGGTGAATCTAGACTTAAAAGATATCGAGAAAGGATACAGGGGAAGCGTGAGTCTGAAAAGTAAAATGCTCATGTTTTGGATTTGGGTCATCGAGTCATTAAATATAATAGTAATCGAAAAATAGTTGAGTCGATACAGTTGAGACGATTGTGAAATGCAACCAAAGATGTGTGTTAGATGTGATTAAATGATGTAATATTAATATATATGTATGAAGAGTTTTTTTAAAAAAATAAAAAGAAATTGGTTAAATATTTTGCTTAAAAATGTTTTATTTGTTGCCATTTCTCAAAATTGAGAGTATGTATTTTAATTTAAGAAAGAAAGAAATAAAGGAGAAGAAAAGAAGAAGGGAAGAAAAAAACAGAGGTTGGTATCAAACAATGTCTACTACTGAGCATCTGGTTCAGTTGGACTGGTCTTAATGGGTACAATTTCCACAGCAGATTGTTTCATATTTTGTCAGATTCAACACATTATTATTATTATTAACCCAATCCCCTCTTCTCCCTCTTCATTCAAATTTCATCTCCTCCCTTTCTCGTCTCCATCTGATTCTCACAACTATTCTTCTTCTTCTTCTTCTTCTCTTTTTAATTTGTTCAAGTTTCTCTTCACTTACAGCCCATCCACTTTTTAATTTGTTCAAGTTTCTCTTCACTTACAGCCCATCCATGGTGATGGATTAATGGTTCCACTCCCAATACCCCCTCCCCCCTTTCTCTCTCTCCCTCTGTGTTTTTTCTGACTAATTTTCGGTCTCTGTGACTGTGACCCATTTTTCTTTTGACCCTTTTCTCCCCTTTCCATTGTAAATGGAATTCAATCGCACGTAAAACAAAAACCCATTGCCCCATTTCGAGACAGTTTTTCTATTTTTTATTCTAAGCTATGGCTGAGGAATATGGCTAAACTATTCACTCCTCTGTTTTTTTTTTCTTTATTCAAATGCCTCTCAGATCCAACTTGTAGTACTTGTTTCACAGTGAGATAGAGATAGATAAAGAGAGAGAGAGAGATATGGGTTTTTTTGGCCGTCAATGAAGCAGTGCCAAGGTAACTTACTGCAACTTTCTGGGTTTCTTTTTTTTCTTCTTTTTGATCTTGGGTTTTGATTTGAAAATGTGATTAAGGGTTCCTGTTTTCTGTTCTTCTCTTTTCATCGAAAACCCATCTTTCGTTTTTGCTCTTAACTTTTTTTGTTTCTTCGATTTTGAGTTTTGGGTTTGGTTCTTTATTGTTTCTGGAGCTTTCCATTTTGTTTTCTGTTCTTCCATGGCGGTTGAACAGAGTAAGGCCCGGGTTTTTCTTTTTTACTGTTGCATTAAATCAGAGTGAATGAGTTGAAAGTTTCTCAAGTTGGATCCGCTTTCAAGTTTTTTAAAGCT

**7. Csa6M181600.1:**

**Promoter：**

AAGTATTTAAAAATGGATATATAATTAGTGATCCAAACTTGAAGGCTTATTAAAATGACACGTGTGAAATTGATTGGTTGAGAAGTTGCATGTTTTGCTTTAAAAGCAAACCACACGTGTGTGTGCTGCCTCCTCGTGATTTTCATCTCACGTGATCACCATACTAGTTCTACCCTTCACTACACCCACCAACTTTTTCCCTCTTCACCTCAATTAAATATTTGATTCTAAATGCCAAACCAACTATGACGAATTTAGTAATTCTAAATATTTGATTAATAAAAAGAAACGTAAGACTCAGATTCAAATAGAGTTGAAAAGGATGAAATTAAAAAGGTTAGAGTGACATAAAGTTATGTATAAATTGACTTAGATTTAAACTCATTATTTGCCTCAAAATTTAACTCCACCTCTCCTTCCTTCCCATGGCGACAAAATGAATAAACAATAACAAAGATGAGCTGGCAAAATTAGCAACTTTATCTTTACAAAAAAAGTCAGAACATAACTTGTCTTTTCTTTTAATATTAATAAAAAATAGCCACTAAATTATATATATATATATATATATTATTCTATACAATTCAAAATTGTACTAACTCACTGACCCAACACCAAACCCAATAAAAAATATATATATTAAAAAATATTTATAAGAAAAGAAAAAAAGTCTGACATGTCCACAATGTTGTTCAGAGAATCTCACAGAGCAATAAAAAAGGCCTTTCCCTGTCCGATCAATGGGCCACCCCCTCCCTTAAGGTGGGCCATGGATTGGACAGTCTTTTTGTTTTCTTAATTGGGCCTAATTTTCAAAGGCCCACTCCTAATCTTTGACTTGCTAAGTCACACCTCGTCTCTACAAATCCAACCCTTGATTTTACCAAGTCAAATTCCATCCACACGTTTTGTTGATGATGATGACCTATCACAAAAGCTTCTATTCCTAACAAATCCGAATTCCCAAATGTTAAGCAATAAACCCATTCGTTTCCTTTCATCATTTCCGGTACTCTGTACAGCAAAACCTCTCCACCCACCACCATCTCCGCCCCACGGAACATCAAACTCACTGATGGCAACGTTGGTAGTTTCCCTCCCGCTGCTACACTATAGCACAAGTCCATCGCTCCTTGGAAAACAAAGTTTGGATCACCCAGTGGAGCCAAAACCCCTTTTGTTTGCTCTAGAAACTCGTTTCTTAAAGCTGTGTACACTGGTCCCAGAAGAAACGTGAACTGGGTCCCTGAATCTACCATCGTTTGCCCGGCGCCGGTGTGATCCGGTGCGAATATTGATTTCGGGAGTGGCAGGATTTTGTTTCCTACCCTGATTCCGTCGAGTTGGACTGTGTAGGCGACTCGGTCGAAATACGGTAATGGGGTTGAGATTTGAACTAAAGGTGTGTAGGTTAAATTCCCAAGCCATGAAAGATGTAAGTCGCCGAAGAGAAGAACCCCAGAAGAATCACGACCGGATATGCAATAAGAGAATTTGGGTAAACCCAATTGGGTAACAAACGACAGTGATCCTCTGTTCATGCCCATCAATCCAGTGGTTTTCGCGTCCTCCTCCGAATTGGAACTGAAACCCGAATCCATACACCCGAATAAAGTTCCGGGTAGAGCCGATGATCCGATTCGAAAATTGTCTGATGCGAGATTTCCCTCGAGTGAGGACGCATCGGCGTAAGAGACAATGGCGTGGCAGAGCTTTTTCGGGTCGCAGGTAACCGGGTTGGGTAAATCTCGGGTCCGGGTACGGCAAATGGGGGAAGAACAGGGTATTGGTGAGTAAGAAGAAGAAGAAAGTGGGTTAAAAACAGAGGTCAAATTTGGGGATTTCTTGCAGTGAAGCCATGAGAGTTCACTGCCTGTATCAAGAACCATGGTGACTTGTTGAGGAGGTGAACCAACGGTTAAAGAAACAGTCAAAGTGACATTATGGTGAAAACTAAGCTTGTTGGAAGGCTGAGAAATCAAACCCATCTGAGTGTTGAGAGGCAAAACC

**8. Csa6M344280.1**

**Promoter：**

ATTTATAAAATACAAAATACTATAATATGCTAATTTTTCTAAAATCTCTTTAAGAATCTTTTATTTAAAAATAAAATAAAAGAAAGGAAGGTCCCATGTTTAACATGGGACATAGAACAATACTAGATACCCCTCCCCCCTCCATTGTCCTTTTCTCACTTTCCAAAAACATCATTGTCTCTATTTTTTATTCATTTTTCTAAAGGTATTTACTTAGATTAAACTTTGTAGTTTGGGTAAAAAAAAAAAAAAAATCCTATGCGATAGGAACTTTACGAGGAATACTTACACAAAAATGTCCTTAAACTTTCAAACAGAATATACCCAAATAAGTATTGTATGCGTTATGAGGAATGTATTCCTATGCGATAGGAACTTTTATGAATGATTTGAAAGGAAATGATTTTACTATTTGAATGCGTAACTATTAATATAGCATGGTTTTGGAAAAAGAATAGCAACACACGTTATGGTTAAAATGTATTTACTAAATATGTTGGTGAAAACCCCCACTCACGAGACACTTTTGTTTAATATTTCAAATGTTTTATTTTATTTTTCCCTAGATAGCGAAGGACGTTCAATGTTAGACTTGCCAAATTGAATGTCTGCCATGACATAATTGCGTTGCATCAATTTGTTAGTATTTTCAGTCAATTCACATCACATGAAGTGTCCTAGGCGAGATGATCAAGATTATTGTTTTTGTGACATAACAAATCAACAAATAGTGAGTAACCCACACTATTAGAAAAGCATCCTTGTTTGTTTCAAGAATTCTAGAGAGTCCATACAAATAATTAGGTTTAAATCCTATATTGGTTCCTAAATTTTGTATCTTGTTTTATTTTGGTCCCTAAAGTTTTACAAGTGTTTGTTTTAGTCTTTGAACTTTTATAAAGAACGTATTAAGGTTTTTACCGTTAAGATTATGCTAACAATTTAACAAAATGATGAGGCGACTTCTATATTTGGATGACTATGATGCCAATTAAGATGACCACTTTTAATCATCTATGATTTTAAGTTTTACCTAATGTGGAACAAGATACAAAATTTAGTGACTAAAATAGAACGGTAGCTAGTTAAAAAGTTAAGGAAGTGTTTGTGGTGGAAGAAGGGAGAATGAAAAGGGGGGTATGATAAAAAGGTAAAGGAGAGGGATTTAAAAAGAAAAGGTGCATTTTGGTTTGTGGATGAAATGAAAAAGGAATTGCTTTGGAGTGCGGCTTCGGTTTTGAACACATCGAACCTTCTACACACACAAACAACTCAGACTCTATTTTCCCAAATAATACAAAATGAAATTAATCTTTTTAATAATAATAAAAGTAGAAAGGATATATATTTTTATTTTTTTTATCTTTTCAAAACTCAATAAAATGGATCCTTTCCTTTTCCTACGCAAAAAGACGAAAGTGGGTCCCATTGAAACTCCCGAAGCCCATCCTCACACGCACGCTTCATGCTTTCCACGTGGCTCAATCTCCATTGCCCTTTCTTTGCCACCGGACCTTTTCTCTTTCTCCCCCTCTCTTTCTCTCTTTTGCGTGTCTTCTATTTTATAGACCGTTTCACCTCTTTTGCTTACGGGAATCTCTTTGTATTCATCTTCTATCCTTCCCAGGTAATCGCTCTCAATTCCTTTTCTTCCTTTTTGTTTCCCCCTAAGCCCTTCTTCTTTTTCTCTGATTTTGATCTTTTCTTTTTCCTACCTTCTCTTCTTTGTATATTTTGCGGGTTTCTTCTTTGTTTTCAACCATTCGGACATGGAAGTACCACTCTATTTTCTTTTCATCTCGGTCACTCTCTTTTCTGCCTTTCCTTGAAACCAAACAGCGTACCATCAGGGTTGTAATATTGTTACACTCTTCTCTTTCTTACTGTGCTTTTTTATCTTCCCTTTCTTTGTGTCAGGCAGTAGAAGCGACGAGATTCTGATATTTTGAGCCTGCTTTGGTAAGTTTTAGGTTACGGGAGGTTTGGGGGATTTGAGTGAA

**9. Csa6M490170.1:**

**Promoter：**

ATATACTTCATTTATATATACTCTTTATTCTTTGGTAGGCTGTTAGTAAAAGAAATAGATTTTGTGAGAAAGTAATGGAGCTTCCATCAGTAATGTCGACGCCGGTGACCGGGTTAGTCGCCGCACTGGTGGTTATAGGCTTGACCTACTTGGTTCATAGATGGAGAAATCCTAAATGTAATGGAGTTCTTCCTCCGGGCTCTATGGGCTTCCCTCTCATCGGAGAAACCCTTCAGCTCATTGCTTCCGGTTACACTCTTGACCTTCCTCCCTTCATCAAGAAAAGAGTTCACAAGTAATAATTGTTCTTTACTATTCTTTTTTTTTTTTTTTTCAATCTCTGTTTCTCTTATATGTTATTTATTATTTGCCAGCAAAAATTTGTTTAGCTTTTTGGGATCTTTGATTTGTATGTTTTTGTTAGTTAATTCAAGTTTAAGTTAAAAGTAATTTTAACAGTTCAAATAATTAAGATCACTTTTGAAAGTAAACCATCTTTATTCAAACAAAAAACACTTCTCAACGTCGAAGCTAAATCCAAAATGTAGGAATAAGTTAGCAAAAGGTTTCTTTTTTCTTATGCACTTATTTATATTAACATTAAGTATTGTTCTTGGGGGGATTTGAATAATGTGCAGATATGGACCCATATTCCGGACGAGTTTGGTGGGTCGATCCATTGTGGTAACAGCTGACCCTGAAATCAACAGTTTCATATACAACCAAGAAGGAAGGACTGTGGAGCTTTGGTATCTAGACTCCATCTCCAAGGTGTTTAAGCAAGACGGAGAGGTTAAAACCACCGCCGGTGGAGCCATCCATAAGTACCTTCGAAGCATCACTTTGAACCACTTCGGTTCCGAAAGTCTCAAGTCCAAGCTACTGGCTGATATCCAACGATATGTTGATAAAGTTTTCACCCAATGGTCTAACCACCCCTCCGTAGAAATGCAACGTGGAACTCTTACAGTAAGTTTCGAGTAAAAAAAAACCAAAATAGCTACTAAACGAGAGTTTAAAATACTAAACTAACTGTATTTATATATTGTTGTGTTGTGTGCAGATGTTATACGATTTCAATGCGTATATAATGTTCGGTTATGACCCTGAAAAGTCTAATGAGAATATAAGTGAGAGCTTAATCACATTAGCTGATGGCTTCATGTCTTTCCCAGTGAACGTCCCTGGAACTAAGTATAACAAGTGTCTTAAGGCACAAAAGAGGTTGGTCAACACGTTCAAGGCTCTCGTCAAAGAGAGGCGCCAAGCCTCTGTTGCTGCTGCTCGTGGGGATTTTCTCGATCAAGCCCTTCGCGACATTGAAAACGAACAGTTTCTCACTGAAGAGTTTGTTTCCAATTTGTTGTTTGGTGTTTTATTTGCCAGTGGCTCTATTTCTGGATCTCTTACCCTGATGTTCAAGTTACTTGCGGAAAATCCATCGGTCGTGAAGGAGTTGACCGTAAGTAATTATTGAAAGTATTTTAAAACATTTAGAAAATCAATCTTCAAACATATATTTGTAATTTTGGATAAAGTAACTAAAAAATATACATGGACAGGCTGAGCATGAGACATTCTTGAAACAGAGAAAAGATCCAAAATCTCCCATCACATGGGAGGAATACAAGTCAATGACATTTACGCTTTACGTAAGTTATAATTATGATAAACGTAACGTTGTTTACCGTTGAAGTGGTTGTATGTATATGTAATTATGTTGCACATGCATGCACAGGTTATATACGAAGTTTTTAGATTATCAAACGCAATGCCTTTTCTGTTGCGGAGAACTACAAAAGATGTGAACATAAAAGGTAAGTAACCATTTTGGATTTAAATTAAAAATTTTGTATAATGCATATGTTATATGTATCAATCTGTATGATGTTGAGTTGGTCGTGAAAATACATGCAGGATATACAATTCCAGCAGGGTGGACGATAATGGTTGCCAATTCAGCTCTTCATTTGAACCCTCAAACCCACAAGGATCCCTTGGACTTCAACCC

**10. Csa7M425920.1**

**Promoter：**

TTTATTTGGATTGTAACTCTTCATTTAAGATGAATCTTTGCCATAGAAGTTGCTGAAATCTTGTTCTTGTTATTGGTTGAATTGTTATATAGGATTTGGAAACAAAACTATTTGTTGTATTTATCTTCTTGAAGATTGTTTTATCCTATGGATGGTTTGTTTGTTTTATGAAATCATGCTCTTGTTATTTAGGATTTGGAAACAGAACCATTTGTTGTATCTTTTTTAGATTGCTTTATCATTTGGATGGCATGGCGGTTTGTTTTCTGAATCTGTATTTATATTTTCTTTTGGTTGTTTATCAGGCTTGGATCTCTTCTACATGTATGTATTGGTTTAGTCTCCCTAGTGGTAAAGAAAACTCCCTTCACTCTGGCAGCTGCAACTAGCTCCAAGTCTTCTCGCACCTTCAAATGGGTGTGGGATAATGGAGTGATATCTCTTGGTGTTTTGAGGAAATCAAGATCATCACACATTTGGAGATTGTGTCGTGTTTTAGAGGGGCTGTCGCTCGATTACGGCTAAGGTTATTATTGGCTAAACATTGTTGAAACTATCTATGTAGTTGTTCTTCCGATTCGTCTTGTAAGGATTAGACCTTTGTTTTAGGACTTGTCATTAAACGAGCAAGTTGGTTTTTGTTATCGATTTAATATTGTGTACAGCAAATGTAGGCTGTCTGTGGATGTAGTTTTTCAGTTGGTCCTCCTTCTCCACAATGGTTATATTTTCTTGTTTCCATGCTCACAATCATAACCACAAAGTAAAGGTAAATGGAAAGACTCACTTCTGGACATTGACGTATATTGCTTCAATTAATACAGGTTGTGTATAATTGATTTCGTCTGTCCGTGTCATATACAGAAAATGGGCCAACAATCTCCCGAAGCAATGCAGAAATCACTTCAAGATCTTTCTCAAAGTAATGTCTCAAAGAATGCTCCTCAGAGTACAACTCCAAATCAATTATTGATAAAGACAGGGAATGCTAAACAAGATATCAACAGAGCATATCACATGACTTCCATCATTGAGAATAGCAGAATGGCTAATGAGACGTGTCGCCTGAAAAAAAGTCAGTCCCTAGGAAGCATGCCATATGGGGATGGTTTGGCTGGTGCAGACAACGACACTGAAGAAGGCAGAGTACTTTCCTGTGACCGTTCCCAATGTAGGCTTGAGATTTCAGATTCTGAAAAGGCTCGAGGATTGGGCATCTCTGATCGGTTTAAAGACACTGTAGCCTCGGATTCTATTCGTGCAAATTCTGGTGCTGTTATTAAAGAAGAAATCTTCTCTATCGATGACCCGGCATGTAGAGAAATGGAAGGTGGGGATAATGCTGGTTCTATGCTATCTTTTGATGGTGATAATAGGAATTATACACCTAGCACCACACAAATGATTGTAAAATCATGTTCAATGCCGAACTTTGATGCCTCATCACCTGTTTCTGGTGGTTCTCCCTGTAAGGATTTCCTACCACCATCGAGATCTTCTGATGATCTCCAGCTCTTTGTTCCAAGGCATGGAGAGATCTCACTTCATGAGATGGAAGTCCAAGTAAATGGATCGCAATCAAGAGAAGATATTGTGCATGAGAATGAGAAAATTTATTATGAAAATTTTTCGGATGACGGAAAAGATTCTTACCACGATGTTGAGCGGGACTGGAAAACATCAGTTGTAGATGAAATAAATCCAAGGGAGGCTCTACAAGAAGAATCTGAATCCCCGGTATATTATCTGAATGAATTGCCAACCAATGACTTCAGGATGAAGCGTATTGAGGAGTGGGTCAGTGATCTTCAACATTGTAATTCGTGTGATGAAACAACTGAAGTATATGAATCTGCTGTTAATGAAGTGAAAAGAGATTCTAGTATAGAAACTGGTTCCAGTGTTGGACGAGTCGATTCCAAAGTGACTGCAGGCATGGAAGCTGCTAAGAGATATATATCTTCAATGAATGCAGCAGCCACTACTGCACAATTGGCAAACC
