# Supplementary material for: Transcriptome Profile Analysis Reveals that CsTCP14 Induces Susceptibility to Foliage Diseases in Cucumber
Source: Int J Mol Sci. 2019 May 26;20(10):2582. doi: 10.3390/ijms20102582 (PMC6567058; doi:10.3390/ijms20102582)
Supplement: Supplementary file 1 [file ijms-20-02582-s001.zip › ijms-505688/Supplementary Files/Supplementary Table 1.docx]

**Supplementary Table 1.** Information of 27 CsTCP gene homologs in the cucumber genome.

| **Gene name** | **Gene ID** | **Physical location on chromosome** | | | | **Deduced polypeptide** | | |  |  |
| --- | --- | --- | --- | --- | --- | --- | --- | --- | --- | --- |
|  |  | **Chr.** | **Starting position (bp)** | **Termination position**  **(bp)** | **Coding sequence (bp)** | **Protein length (aa)** | **Isoelectric point**  **(pI)** | **Molecular weight**  **(Da)** | **Class** | **Predicted Subcellular**  **Localization** |
| *CsTCP1* | Cs1M009680.1 | 1 | 1434041 | 1435357 | 936 | 311 | 6.18 | 32772.80 | Class 1 | Nuclear |
| *CsTCP2* | Cs1M014310.1 | 1 | 1851574 | 1853321 | 1227 | 408 | 8.95 | 44714.32 | Class 3 | Nuclear |
| *CsTCP3* | Cs1M020890.1 | 1 | 2221849 | 2222877 | 1029 | 342 | 9.17 | 38801.23 | Class 2 | Nuclear |
| *CsTCP4* | Cs1M025920.1 | 1 | 2936749 | 2939005 | 1332 | 443 | 6.82 | 48364.22 | Class 3 | Nuclear |
| *CsTCP5* | Cs1M033030.1 | 1 | 3495021 | 3496981 | 900 | 299 | 7.25 | 32561.23 | Class 1 | Nuclear |
| *CsTCP6* | Cs1M039270.1 | 1 | 3862724 | 3863411 | 540 | 179 | 9.32 | 20394.35 | Class 3 | Nuclear |
| *CsTCP7* | Cs1M042180.1 | 1 | 4128095 | 4129330 | 1080 | 359 | 9.31 | 40912.96 | Class 2 | Nuclear |
| *CsTCP8* | Cs1M077150.1 | 1 | 7879722 | 7880533 | 525 | 174 | 9.54 | 18912.21 | Class 3 | Nuclear |
| *CsTCP9* | Cs3M179190.1 | 3 | 12296806 | 12298588 | 1029 | 342 | 9.49 | 36314.03 | Class 1 | Nuclear |
| *CsTCP10* | Cs3M265270.1 | 3 | 16322418 | 16323535 | 864 | 287 | 9.71 | 32717.98 | Class 2 | Nuclear |
| *CsTCP11* | Cs3M564320.1 | 3 | 22137863 | 22138816 | 891 | 296 | 6.37 | 31669.40 | Class 1 | Nuclear |
| *CsTCP12* | Cs4M088720.1 | 4 | 5770292 | 5773170 | 1287 | 428 | 6.33 | 46383.63 | Class 3 | Nuclear |
| *CsTCP13* | Cs4M132680.1 | 4 | 7986911 | 7988943 | 927 | 308 | 8.95 | 34458.20 | Class 3 | Nuclear |
| *CsTCP14* | Cs4M628330.1 | 4 | 20430639 | 20432861 | 1290 | 429 | 6.28 | 46933.12 | Class 3 | Nuclear |
| *CsTCP15* | Cs5M175170.1 | 5 | 7291445 | 7292933 | 771 | 256 | 9.92 | 26540.55 | Class 1 | Nuclear |
| *CsTCP16* | Cs5M352090.1 | 5 | 13488734 | 13489920 | 795 | 264 | 9.77 | 27381.65 | Class 1 | Nuclear |
| *CsTCP17* | Cs5M577450.1 | 5 | 20393203 | 20394105 | 903 | 300 | 9.22 | 34667.57 | Class 2 | Nuclear |
| *CsTCP18* | Cs5M587110.1 | 5 | 20993270 | 20995045 | 1266 | 421 | 6.76 | 43093.52 | Class 1 | Nuclear |
| *CsTCP19* | Cs5M605000.1 | 5 | 22648606 | 22650181 | 1104 | 367 | 9.09 | 39002.15 | Class 1 | Nuclear |
| *CsTCP20* | Cs5M608320.1 | 5 | 23366150 | 23368300 | 1167 | 388 | 9.03 | 44144.61 | Class 2 | Nuclear |
| *CsTCP21* | Cs5M644520.1 | 5 | 27044242 | 27045612 | 1188 | 395 | 8.50 | 44720.99 | Class 2 | Nuclear |
| *CsTCP22* | Cs6M075180.1 | 6 | 5021969 | 5023249 | 978 | 325 | 8.51 | 34881.04 | Class 1 | Nuclear |
| *CsTCP23* | Cs6M093640.1 | 6 | 6417103 | 6417981 | 879 | 292 | 7.97 | 31543.13 | Class 1 | Nuclear |
| *CsTCP24* | Cs6M147520.1 | 6 | 10195168 | 10195728 | 561 | 186 | 5.57 | 20596.05 | Class 1 | Nuclear |
| *CsTCP25* | Cs6M156050.1 | 6 | 10991617 | 10992573 | 957 | 318 | 8.58 | 35002.87 | Class 3 | Nuclear |
| *CsTCP26* | Cs6M446400.1 | 6 | 20921871 | 20922643 | 678 | 225 | 9.30 | 24188.69 | Class 1 | Nuclear |
| *CsTCP27* | Cs6M524000.1 | 6 | 28172060 | 28177254 | 1350 | 449 | 9.34 | 48972.23 | Class 3 | Nuclear |
