# Supplementary material for: Transcriptome Profile Analysis Reveals that CsTCP14 Induces Susceptibility to Foliage Diseases in Cucumber
Source: Int J Mol Sci. 2019 May 26;20(10):2582. doi: 10.3390/ijms20102582 (PMC6567058; doi:10.3390/ijms20102582)
Supplement: Supplementary file 1 [file ijms-20-02582-s001.zip › ijms-505688/Supplementary Files/Supplementary Table 3.DOCX]

**Supplementary Table 3.** Primers of CsTCP family for qRT-PCR analysis.

| **Gene ID** | **Primers** | |
| --- | --- | --- |
|  | **Forward primer(5’ to 3’)** | **Reverse primer(5’ to 3’)** |
| *CsTCP1* | AGGACACTTAAATATACTCGCCGGA | GACTGGTGACTCGTTGTATCATGC |
| *CsTCP2* | CTTTGTCCAACCCCTTCATGTCTTC | GCATTATTGGTGTCAGCTGCATGAA |
| *CsTCP3* | AAGCTCACCATGGATGAGTAGTACT | CTCCACCTTCCCATGATCAAACAAT |
| *CsTCP4* | GAATCCACACCAATTGCCAGTGTC | CCGGCAGAGATGGTGAAGTTAAGAT |
| *CsTCP5* | CTGCTCCATTGACGCTCCTCC | CGGTGGATTTTGGGTGGCATAGATA |
| *CsTCP6* | ATATTGGAGATGCTGCTTTCAAGGC | GGCTGAATTAGTCTGATCGTCCATG |
| *CsTCP7* | AGGGATTCAGCTTGTTGTTCAGATG | TGAGTAGTAGTACCAGCTGATTCCT |
| *CsTCP8* | ACAGTTCCAGTCTTACCCACTTCAA | CTCCGATATTGCTCTGACTTGGACA |
| *CsTCP9* | GGGAACATCAAACAACCATCAACCA | AATCCACTCGAACCGTTTGAAACAG |
| *CsTCP10* | CGGAAGCTTTTGGATCAGATGAAGG | TAATTGGGGTTGCTATTGGTATCCA |
| *CsTCP11* | CACTCAACAATTGCCTGGTTTGGAG | GAGGTTGCTGGTGGTGATGTTGAT |
| *CsTCP12* | CCACCATCAATCCATCAATCCTCCT | AAGCAGAGGATGGCTTGTCTGAAAT |
| *CsTCP13* | CTTTCCACCCAAATGTTAGGCCTTT | TTGATGAGACGCAAATCCATGATCC |
| *CsTCP14* | CTAAGTCCACCGTCGATCTACCAAT | GAAGCAGAGGATGGCTTTTCAGAAA |
| *CsTCP15* | CTAGACCCGATTATGGCCAGGTTTG | CGCCGACGCCTCTCTAATCG |
| *CsTCP16* | GTTGTTCGTTCAGCAGCAATCGA | TCATTGTCTCTTCGTCCGGAACTTC |
| *CsTCP17* | ACAAGATCAAAAGCAGGACGTTACG | TGGAAGAAGTAATAGGAGGGTTTGGT |
| *CsTCP18* | ATGTAGGGAATTCTTCTGGAGGAGG | CCTCCAGGTATATTAAATCTCGGCA |
| *CsTCP19* | TTTCATGAATCTGCCACCACCAATG | CGGCATCGTTGTTCTATAAGCACTT |
| *CsTCP20* | TCTTCTGCCTGATTCTTCCATGGAT | TCTCCATAGAAGATCAACACACCATCA |
| *CsTCP21* | TTTCCAGCTTCCAATTTGACTCCAA | GCTTTGCACACACTTGAAATTCTGG |
| *CsTCP22* | CGCTTCCAAATCTCAATCCGTCATG | AATGTTTTGACGACGAGCTTGACAT |
| *CsTCP23* | CAACAAATACCTGGGTTGGAGTTGG | CTGTGCAGAGGAATCACGATTTTGG |
| *CsTCP24* | GCAAGCCTTGTGATCCATCTTCATC | GAGACCAAATCGAATTCATAGTTTGGC |
| *CsTCP25* | AAACGCCATCCAAATCCAATTCAGG | GACTTTCCACCGTTGTCCTTGC |
| *CsTCP26* | AGTACTCTGCTGCTGGGAATGATTA | CGAGAGGAAGGAGTACGAAGAAGAG |
| *CsTCP27* | AACTTTGCTATGGAGGCGGGAA | GCTTCTAGTCTTCCCTTCTTTCCCT |
| *Actin* | GGCAGTGGTGGTGAACATG | TTCTGGTGATGGTGTGAGTC |
