# Supplementary material for: Transcriptome Profile Analysis Reveals that CsTCP14 Induces Susceptibility to Foliage Diseases in Cucumber
Source: Int J Mol Sci. 2019 May 26;20(10):2582. doi: 10.3390/ijms20102582 (PMC6567058; doi:10.3390/ijms20102582)
Supplement: Supplementary file 1 [file ijms-20-02582-s001.zip › ijms-505688/Supplementary Files/Supplementary Table 4.DOCX]

**Supplementary Table 4.** The primers used in CsTCP14 subcellular localization.

| **Name** | **Sequence (from 5’ to 3’)** |
| --- | --- |
| GFP-TCP14-F | GAGCTCCACCGCGGTGCGGCCGCATGGGAGAGTTCAGTAATCAACGCCCG |
| GFP-TCP14-R | TTCCTGCAGCCCGGGGGATCCATGGCGGGAGTTGGAGGAAGCAGA |

The underline in GFP-TCP14-F was Not I site, the underline in GFP-TCP14-R was BamH I site.
