# Supplementary material for: Transcriptome Profile Analysis Reveals that CsTCP14 Induces Susceptibility to Foliage Diseases in Cucumber
Source: Int J Mol Sci. 2019 May 26;20(10):2582. doi: 10.3390/ijms20102582 (PMC6567058; doi:10.3390/ijms20102582)
Supplement: Supplementary file 1 [file ijms-20-02582-s001.zip › ijms-505688/Supplementary Files/Supplementary Table 6.DOCX]

**Supplementary Table 6.** The primers used in EMSA.

| **Name** | **Sequence (from 5’ to 3’)** |
| --- | --- |
| probes-F | TTGTTTTATTTTGGTCCCTAAAGTTTTACA |
| probes-R | TGTAAAACTTTAGGGACCAAAATAAAACAA |
| Mutant probes-F | TTGTTTTATTTTGGTAAATAAAGTTTTACA |
| Mutant probes-R | TGTAAAACTTTATTTACCAAAATAAAACAA |

The underline represented the binding site.
