# Supplementary material for: Transcriptome Profile Analysis Reveals that CsTCP14 Induces Susceptibility to Foliage Diseases in Cucumber
Source: Int J Mol Sci. 2019 May 26;20(10):2582. doi: 10.3390/ijms20102582 (PMC6567058; doi:10.3390/ijms20102582)
Supplement: Supplementary file 1 [file ijms-20-02582-s001.zip › ijms-505688/Supplementary Files/Supplementary Table 7.DOCX]

**Supplementary Table 7.** The primers used in the Y1H.

| **Name** | **Sequence (from 5’ to 3’)** |
| --- | --- |
| pGADT7-F | GCCATGGAGGCCAGTGAATTCATGGGAGAGTTCAGTAATCAACGC |
| pGADT7-R | ACGATTCATCTGCAGCTCGAGTCAATGGCGGGAGTTGGAG |
| Bait-F | TAAAAGCTTGAATTCGAGCTCAAGACGAAAGTGGGTCCCATTGAAACTCCCG |
| Bait-R | ATACAGAGCACATGCCTCGAGCGGGAGTTTCAATGGGACCCACTTTCGTCTT |
| Bait-m-F | TAAAAGCTTGAATTCGAGCTCAAGACGAAAGTGGGTAAAATTGAAACTCCCG |
| Bait-m-R | ATACAGAGCACATGCCTCGAGCGGGAGTTTCAATTTTACCCACTTTCGTCTT |

Finer lines represented the cleavage sites, and thicker lines represented the binding sites.
