# Supplementary figures and images for: Transcriptome Profile Analysis Reveals that CsTCP14 Induces Susceptibility to Foliage Diseases in Cucumber
Source: Int J Mol Sci. 2019 May 26;20(10):2582. doi: 10.3390/ijms20102582 (PMC6567058; doi:10.3390/ijms20102582)

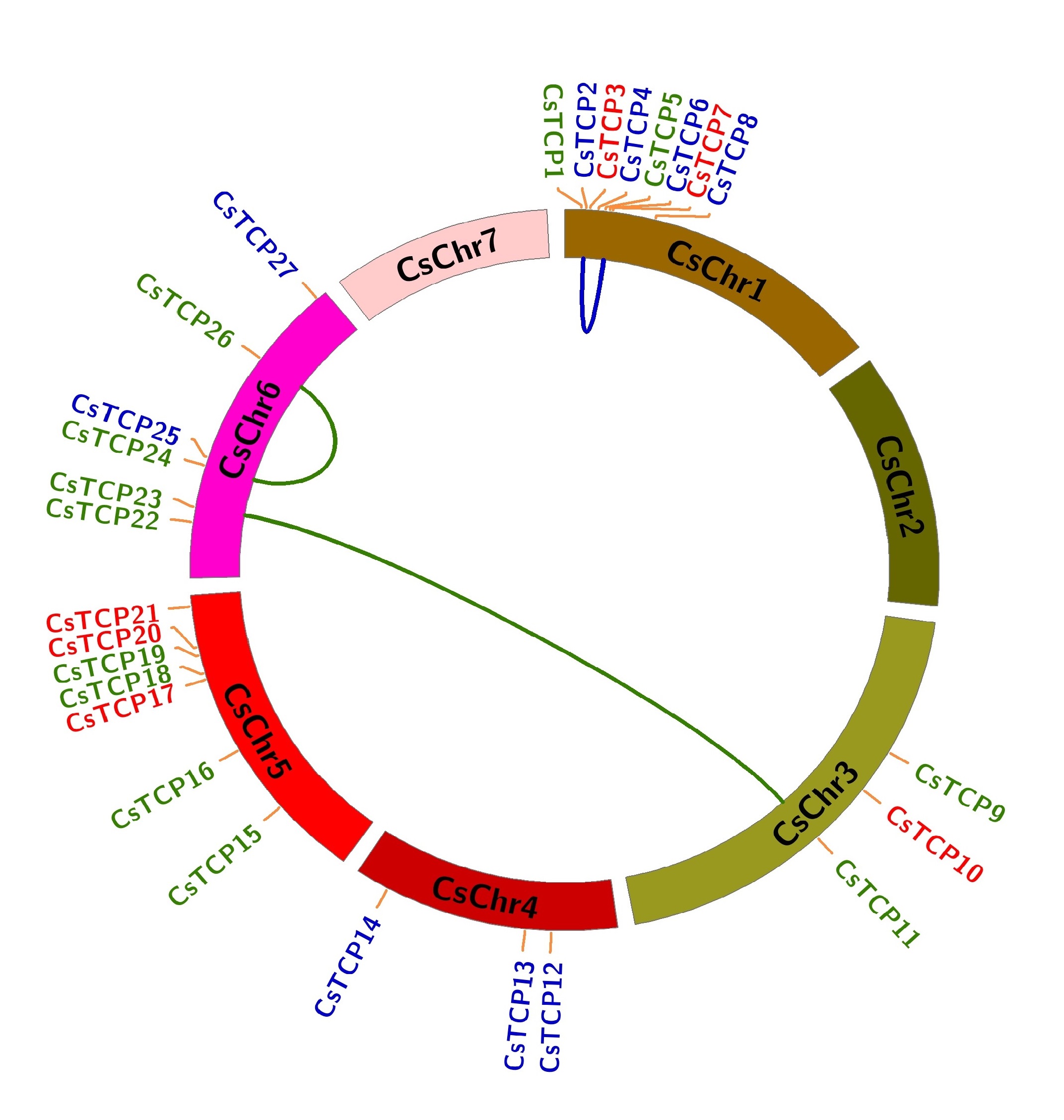

Supplement: Supplementary file 1 [file ijms-20-02582-s001.zip › ijms-505688/Supplementary Files/Supplementary Figure 1.jpg]

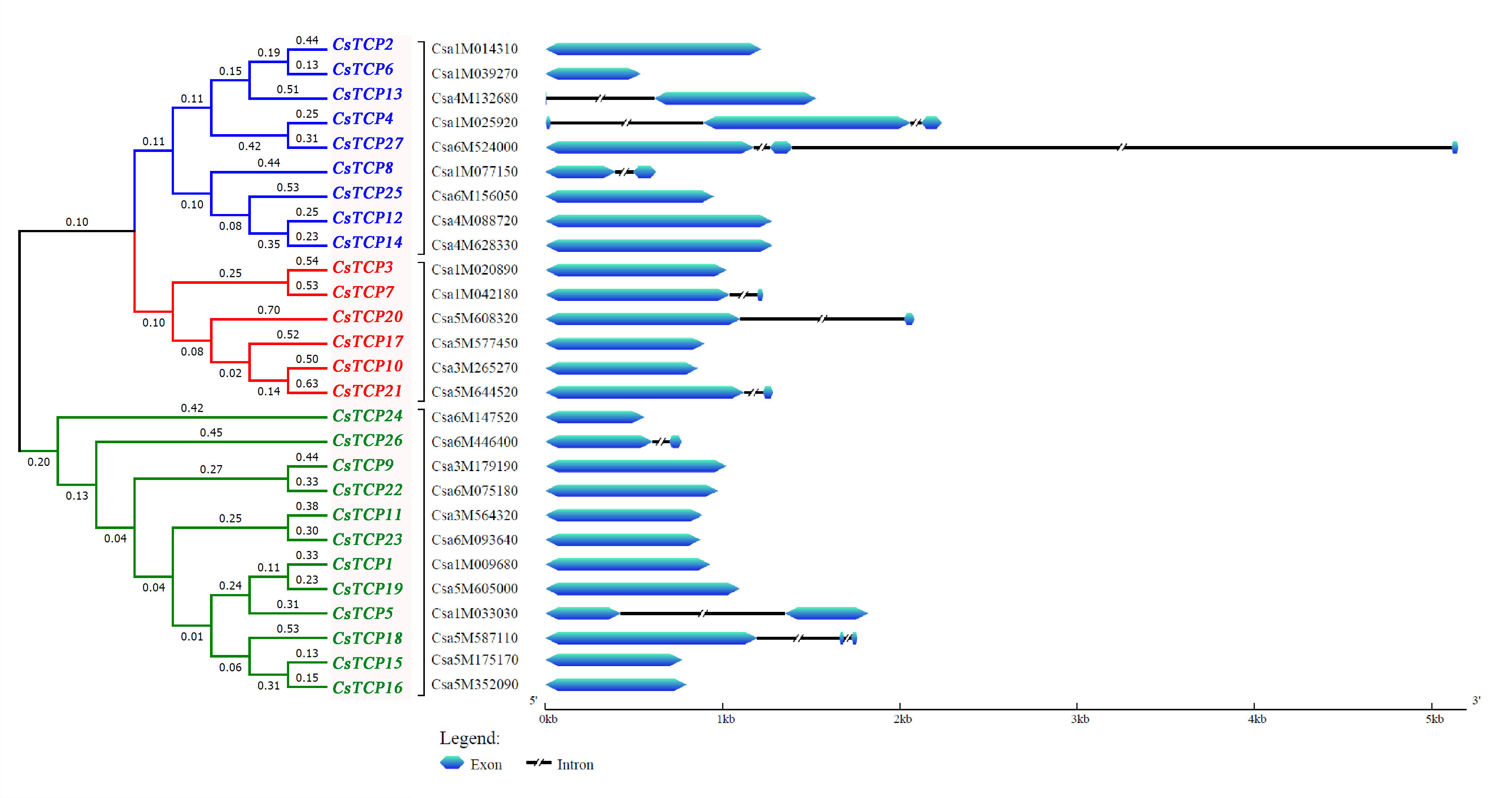

Supplement: Supplementary file 1 [file ijms-20-02582-s001.zip › ijms-505688/Supplementary Files/Supplementary Figure 2.jpg]

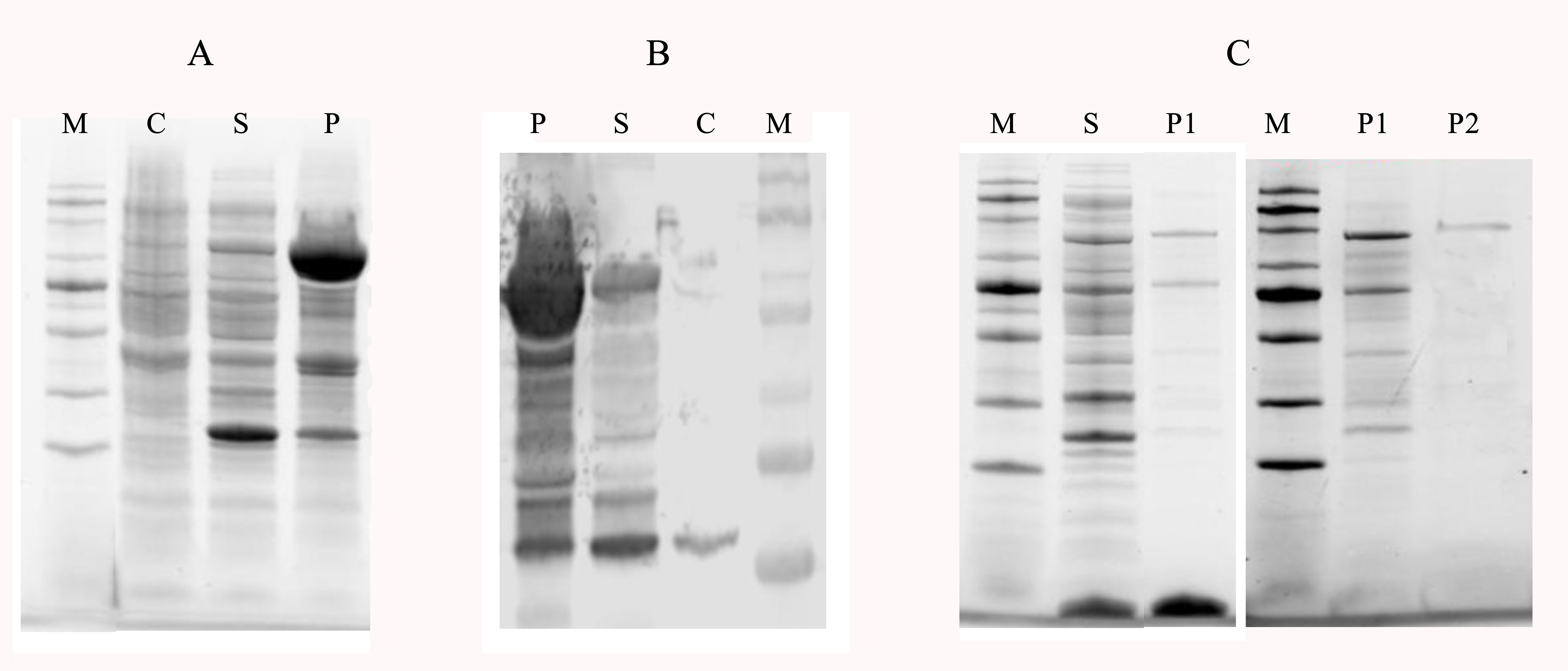

Supplement: Supplementary file 1 [file ijms-20-02582-s001.zip › ijms-505688/Supplementary Files/Supplementary Figure 4.jpg]
